# Study on the Lateral Carrier Diffusion and Source-Drain Series Resistance in Self-Aligned Top-Gate Coplanar InGaZnO Thin-Film Transistors

**DOI:** 10.1038/s41598-019-43186-7

**Published:** 2019-04-29

**Authors:** Sae-Young Hong, Hee-Joong Kim, Dae-Hwan Kim, Ha-Yun Jeong, Sang-Hun Song, In-Tak Cho, Jiyong Noh, Pil Sang Yun, Seok-Woo Lee, Kwon-Shik Park, SooYoung Yoon, In Byeong Kang, Hyuck-In Kwon

**Affiliations:** 1 School of Electrical and Electronics Engineering, Chung-Ang University, 84 Heukseok-ro, Dongjak-gu, Seoul Korea; 2 Research and Development Center, LG Display, E2 Block LG Science Park, 30, Magokjungang 10-ro, Gangseo-gu, Seoul Korea

**Keywords:** Electrical and electronic engineering, Electronic devices

## Abstract

We investigated the lateral distribution of the equilibrium carrier concentration (*n*_0_) along the channel and the effects of channel length (*L*) on the source-drain series resistance (*R*_ext_) in the top-gate self-aligned (TG-SA) coplanar structure amorphous indium-gallium-zinc oxide (a-IGZO) thin-film transistors (TFTs). The lateral distribution of *n*_0_ across the channel was extracted using the paired gate-to-source voltage (*V*_GS_)-based transmission line method and the temperature-dependent transfer characteristics obtained from the TFTs with different *L*s. *n*_0_ abruptly decreased with an increase in the distance from the channel edge near the source/drain junctions; however, much smaller gradient of *n*_0_ was observed in the region near the middle of the channel. The effect of *L* on the *R*_ext_ in the TG-SA coplanar a-IGZO TFT was investigated by applying the drain current-conductance method to the TFTs with various *L*s. The increase of *R*_ext_ was clearly observed with an increase in *L* especially at low *V*_GS_s, which was possibly attributed to the enhanced carrier diffusion near the source/drain junctions due to the larger gradient of the carrier concentration in the longer channel devices. Because the lateral carrier diffusion and the relatively high *R*_ext_ are the critical issues in the TG-SA coplanar structure-based oxide TFTs, the results in this work are expected to be useful in further improving the electrical performance and uniformity of the TG-SA coplanar structure oxide TFTs.

## Introduction

In the last decade, amorphous indium-gallium-zinc oxide (a-IGZO) thin-film transistors (TFTs) have attracted considerable attention due to their advantages such as a high field-effect mobility (*μ*_FE_), a low off-current, and a small subthreshold swing^1–8^. In addition, the a-IGZO TFTs are fabricated at low temperatures (below 300 °C) with a good uniformity over large areas^9,10^. These excellent properties make the a-IGZO TFT a promising candidate for the backplane element of active-matrix liquid-crystal displays and active-matrix organic light-emitting diode (AMOLED) displays^11,12^. Up to now, the a-IGZO TFTs have been fabricated with several structures including a bottom-gate etch stopper structure, a bottom-gate back-channel-etch structure, and a top-gate self-aligned (TG-SA) coplanar structure^7^. Among them, the TG-SA coplanar structure has many advantages compared with bottom-gate structures, such as smaller parasitic capacitance, better channel length scalability, and better process controllability^13,14^. Owing to these merits, the TG-SA coplanar structure a-IGZO TFT is desirable especially for high-resolution AMOLED applications^15,16^. However, despite such advantages, there still remain some issues to be solved in TG-SA coplanar a-IGZO TFTs. One of them is the threshold voltage (*V*_th_) dependence on the channel length of the device^17–19^. In the TG-SA coplanar a-IGZO TFTs, the source/drain extension regions are n^+^-doped in order to lower the source/drain series resistance (*R*_ext_). The high-density free carriers in the source/drain extension regions diffuse into the IGZO channel layer during the subsequent annealing process, which increases the carrier concentration of the channel region and shifts *V*_th_ to the negative direction especially in the short channel devices^17–19^. Therefore, the study on the lateral carrier diffusion and *R*_ext_ is very important in the TG-SA coplanar a-IGZO TFTs to further improve the electrical performance and uniformity of the devices. In this work, we extracted the lateral distribution of the carrier concentration in the TG-SA coplanar a-IGZO TFTs by using the paired gate-to-source voltage (*V*_GS_)-based transmission line method (TLM) and temperature-dependent transfer characteristics data obtained from the TFTs with various channel lengths. Furthermore, we investigated the effects of channel length on the *R*_ext_ of the TG-SA coplanar a-IGZO TFT using the drain current-conductance method (DCCM).

## Results and Discussion

**Figure 1:**
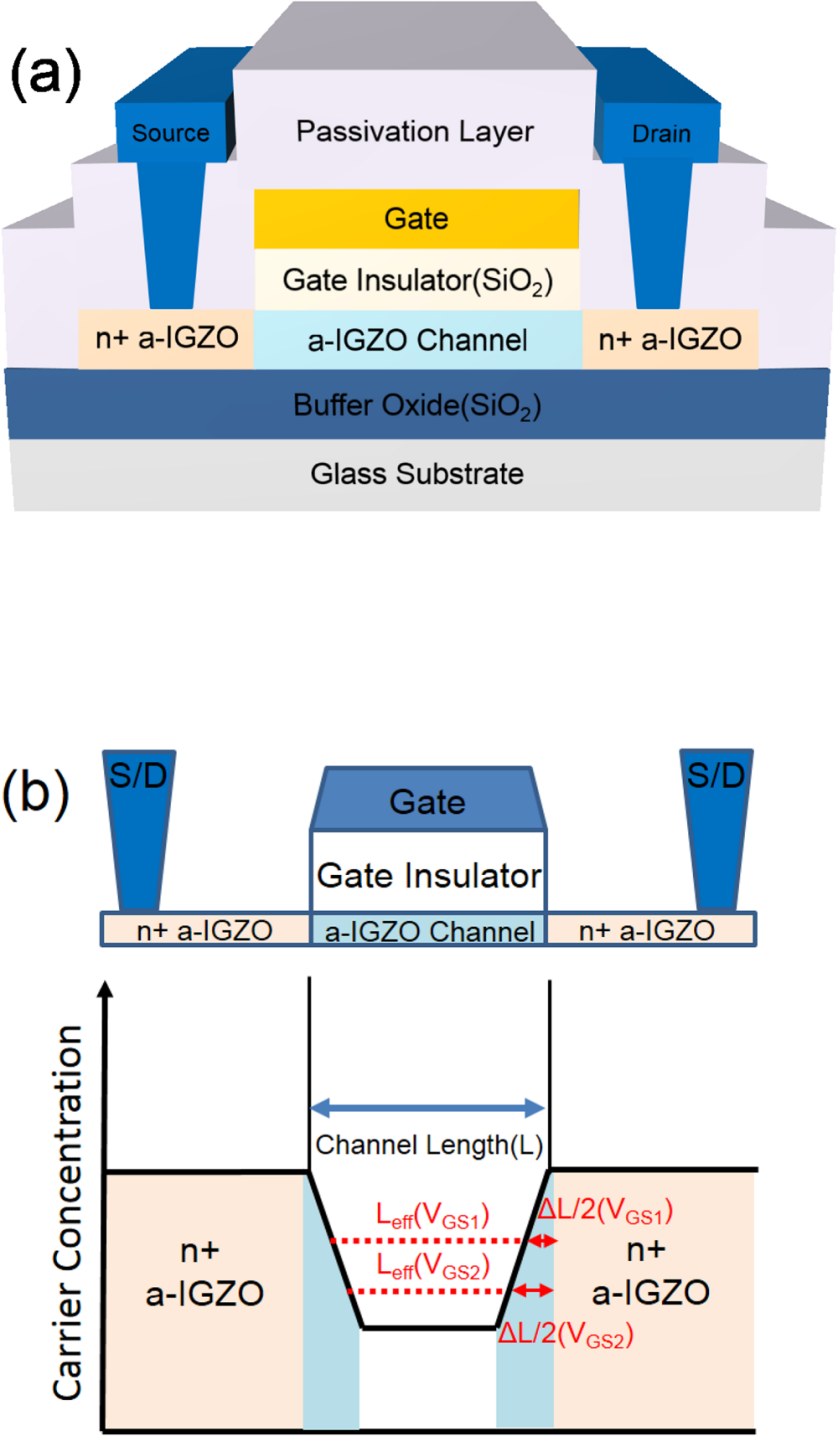
(**a**) Cross-sectional schematic of the fabricated TG-SA coplanar a-IGZO TFTs. (**b**) Schematic carrier concentration plot along the channel between the source and drain in the TG-SA coplanar a-IGZO TFTs.

Figure 1(a) depicts a cross-sectional schematic of the fabricated TG-SA coplanar a-IGZO TFTs, where the fabrication process of the TFTs is introduced in the Methods Section. Figure 1(b) shows the schematic carrier concentration plot along the channel between the source and drain in the TG-SA coplanar a-IGZO TFTs. Near the source and drain junctions, the carriers diffuse from the n^+^-doped source/drain extension regions to the channel region. Solid lines represent the equilibrium carrier concentration (*n*_0_) and the dotted lines represent the *V*_GS_-induced carrier concentration at two different *V*_GS_s (*V*_GS1_ > *V*_GS2_). As can be observed in Fig. 1(b), there are two distinct regions: one is the region where *V*_GS_-induced carrier concentration is higher than *n*_0_ and the other is the region where *n*_0_ is higher than the *V*_GS_-induced carrier concentration. The conductivity in the former region is controlled by *V*_GS_, thus this region can be considered as an effective channel region. Because the effective channel ends where the *V*_GS_-induced carrier concentration is equal to *n*_0_, the effective channel length (*L*_eff_) increases with an increase in *V*_GS_. Δ*L* is defined as the difference between the drawn channel length (*L*) and *L*_eff_ (Δ*L* = *L* − *L*_eff_). *R*_ext_ is the source-drain series resistance associated with all the regions outside the effective channel region.

**Figure 2:**
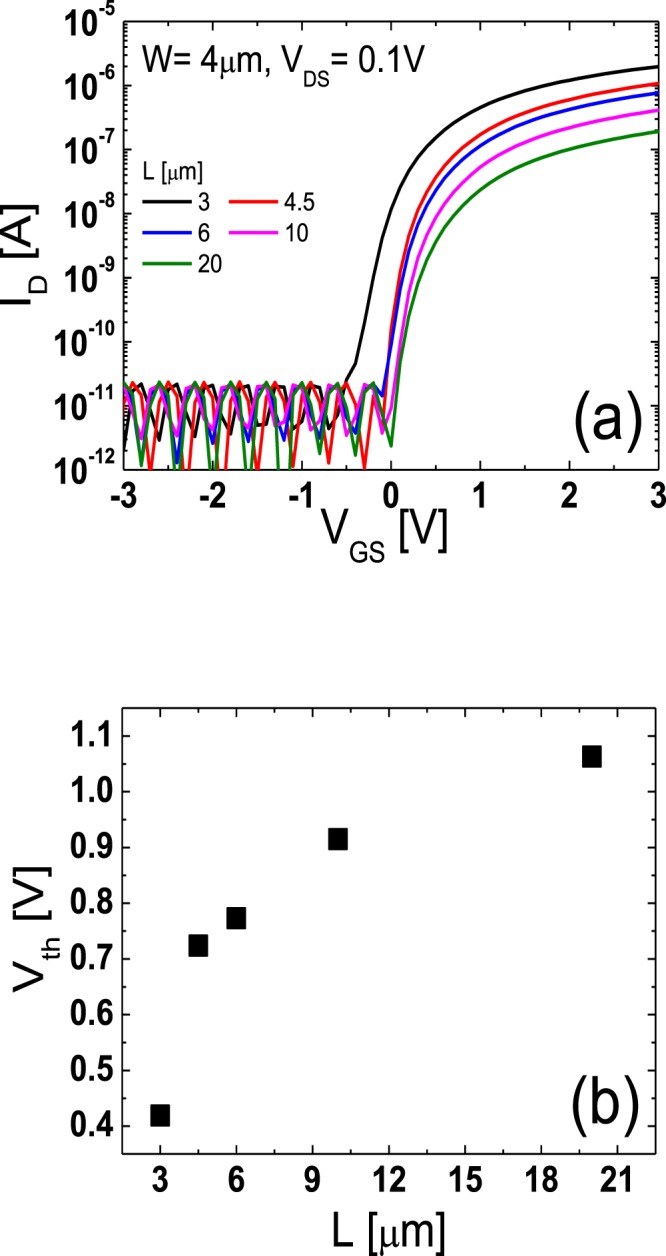
(**a**) Transfer curves of the TG-SA coplanar a-IGZO TFTs with various *L*s (*W*/*L* = 4 μm/3, 4.5, 6, 10, 20 μm) measured in the linear region (*V*_DS_ = 0.1 V) (**b**) *V*_th_s obtained from the fabricated TG-SA coplanar a-IGZO TFTs with different *L*s.

Figure 2(a) depicts the transfer curves of the TG-SA coplanar a-IGZO TFTs measured in the linear region (drain-to-source voltage (*V*_DS_) = 0.1 V). *L* was varied from 3 to 20 μm, while a channel width (*W*) was fixed at 4 μm. Figure 2(a) shows that *V*_th_ shifts negatively and the on-current increases with a decrease in *L*. These results are consistent with those in the previous works^17–19^ and more negative shift of *V*_th_ in the shorter channel TFT was attributed to the higher carrier concentration in the channel region caused by the carrier diffusion from the n^+^-doped source/drain extension regions^17^. Figure 2(b) displays the *V*_th_s obtained from the fabricated TG-SA coplanar a-IGZO TFTs with different *L*s. Here, *V*_th_ was determined by the intercept of the extrapolated curve with the *V*_GS_ axis in the linear-scale transfer characteristics.

**Figure 3:**
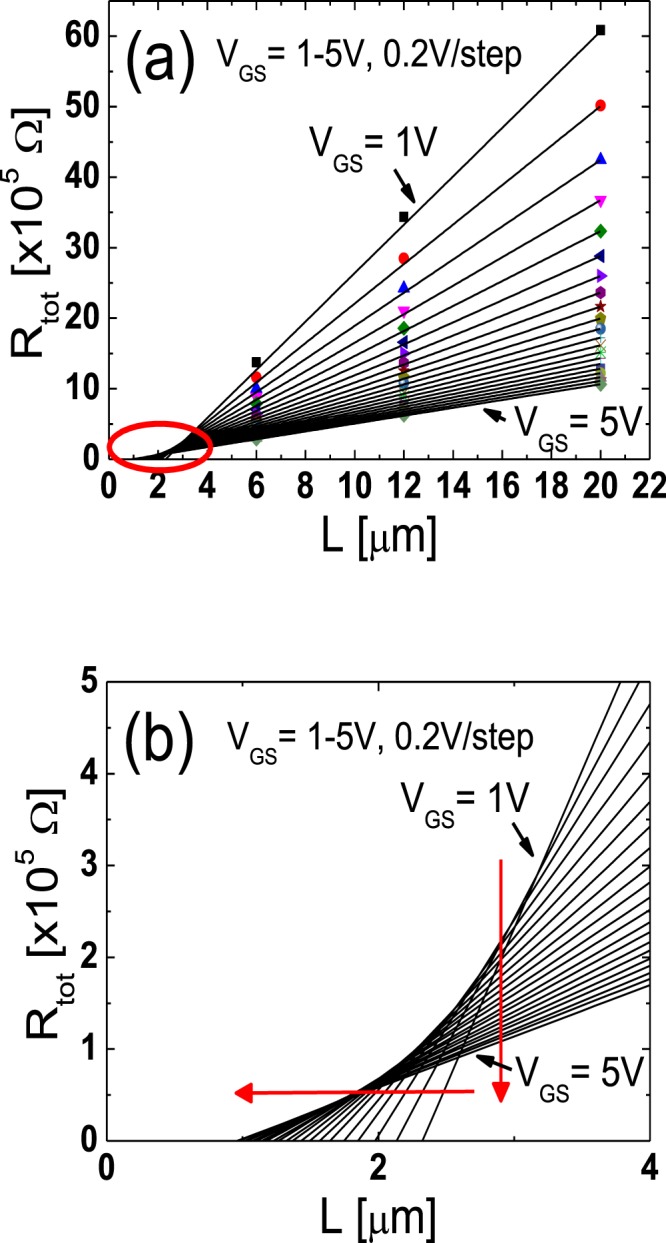
(**a**) *R*_*t*ot_-*L* plot measured from the TFTs with different *L*s (*L* = 6, 12, 20 μm) at a given *V*_DS_ of 0.1 V for various *V*_GS_s (=1 to 5 V with 0.2 V steps). (**b**) Enlarged image of the encircled area in (**a**).

**Figure 4:**
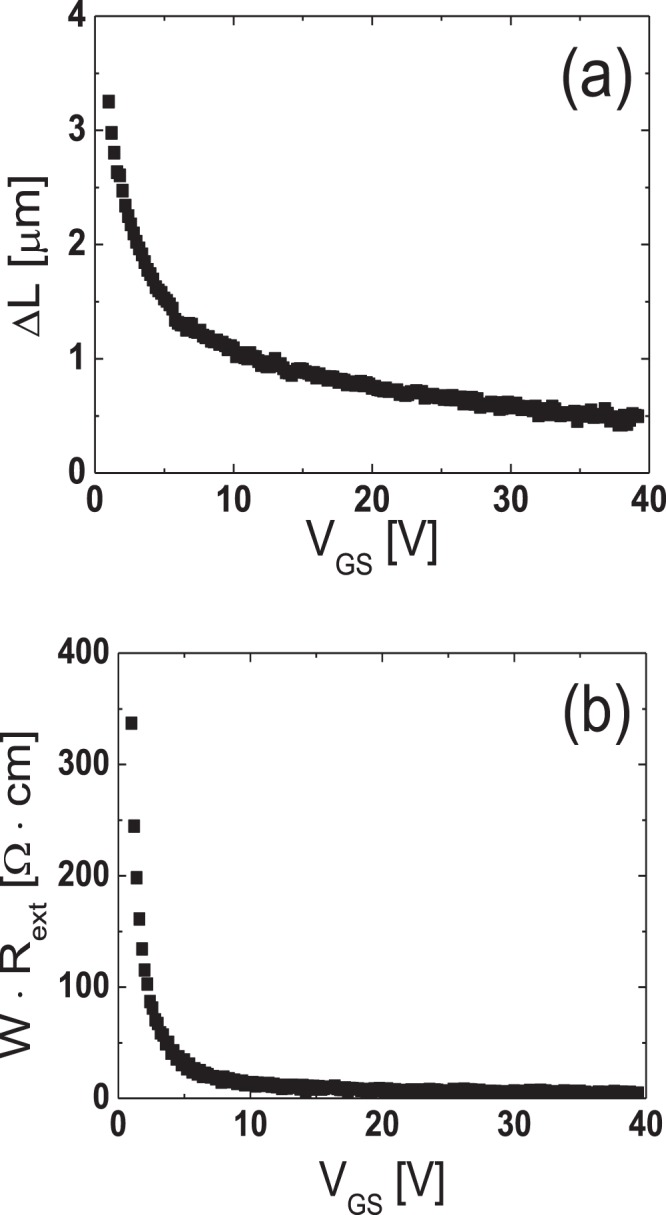
(**a**) Δ*L* and (**b**) width-normalized *R*_ext_ (*W* · *R*_ext_) extracted as a function of *V*_GS_ by using the paired *V*_GS_-based TLM.

**Figure 5:**
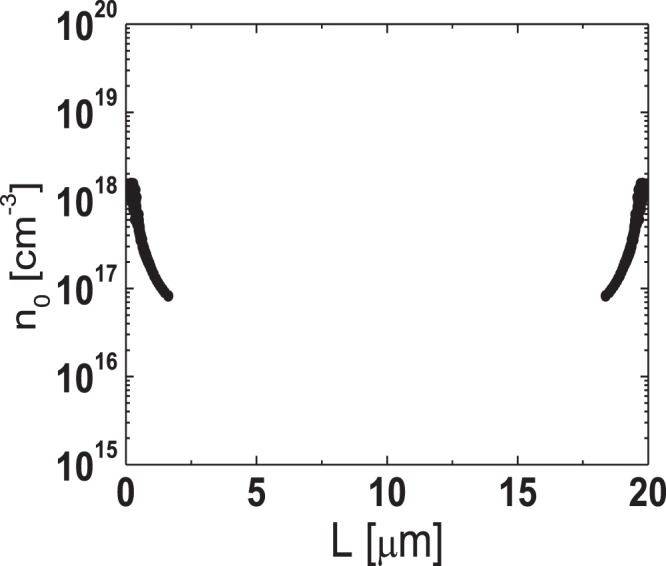
Lateral distribution of *n*_0_ in the unintentionally doped regions near the source/drain junctions in the fabricated TG-SA coplanar TFT with *L* = 20 μm.

To extract the lateral carrier concentration distribution near the source/drain junctions in the TG-SA coplanar a-IGZO TFTs, the paired *V*_GS_-based TLM^20^ was employed. In the TG-SA coplanar TFTs, the total resistance between source and drain electrodes measured in the linear region (*R*_tot_) can be expressed using the following equation:

1 $${R}_{{\rm{tot}}}=\frac{{V}_{{\rm{DS}}}}{{I}_{{\rm{D}}}}=\frac{L-{\rm{\Delta }}L}{W\cdot {\mu }_{{\rm{FEi}}}\cdot {C}_{{\rm{i}}}\cdot ({V}_{{\rm{GS}}}-{V}_{{\rm{th}}}-{V}_{{\rm{DS}}}/2)}+{R}_{{\rm{ext}}}$$

where *μ*_FEi_ is the intrinsic field-effect mobility and *C*_i_ is the gate insulator capacitance per unit area, respectively. Figure 3(a) shows the *R*_*t*ot_-*L* plot measured from the TFTs with different *L*s (*L* = 6, 12, 20 μm) at a given *V*_DS_ of 0.1 V for various *V*_GS_s (=1 to 5 V with 0.2 V steps). Figure 3(b) is the enlarged image of the encircled area in Fig. 3(a). In the paired *V*_GS_-based TLM, Δ*L* and *R*_ext_ at a certain *V*_GS_ are extracted from the intersection of two straight lines obtained at two closely separated voltages (*V*_GS_ ± Δ*V*_GS_) where Δ*V*_GS_ is 0.2 V in this work. Figure 4(a,b) show the Δ*L* and the width-normalized *R*_ext_ (*W* · *R*_ext_) extracted as a function of *V*_GS_ by using the paired *V*_GS_-based TLM. Δ*L* and *R*_ext_ are largely modulated by *V*_GS_, which confirms the presence of the unintentionally doped regions formed by the lateral carrier diffusion from the n^+^-doped source/drain extension regions in the fabricated TG-SA coplanar a-IGZO TFTs. The results of Fig. 4(a) and the definition of *L*_eff_ in Fig. 1(b) allow the extraction of the lateral carrier concentration distribution in the unintentionally doped regions near the source/drain junctions. In the TFT, the *V*_GS_-induced channel carrier concentration (*n*(*V*_GS_)) can be calculated using the following equation^21^:

2 $$n({V}_{{\rm{GS}}})={C}_{{\rm{i}}}\cdot ({V}_{{\rm{GS}}}-{V}_{{\rm{th}}})/q\cdot t$$

where *q* and *t* are the electronic charge and channel thickness, respectively. As explained in Fig. 1(b), *L*_eff_ (=*L* *−* Δ*L*) ends where *n* is equal to *n*_0_ in the TG-SA coplanar a-IGZO TFTs, therefore, Δ*L* is uniquely determined at a specific value of *V*_GS_ by the results of Fig. 4(a). Because both *n* and Δ*L* are obtained as a function of *V*_GS_, we can extract *n*_0_ at a specific position in the unintentionally doped regions near the source/drain junctions by matching the Δ*L*/2 and *n* values calculated at every *V*_GS_. Figure 5 displays the lateral distribution of *n*_0_ in the unintentionally doped regions near the source/drain junctions in the fabricated TG-SA coplanar TFT with *L* = 20 μm (*V*_th_ = 1.06 V). It shows that *n*_0_ abruptly decreases from 1.5 × 10^18^ cm^−3^ to 8.2 × 10^16^ cm^−3^ as the distance from the edge of the channel (Δ*L*/2) increases from 0.16 μm to 1.63 μm.

**Figure 6:**
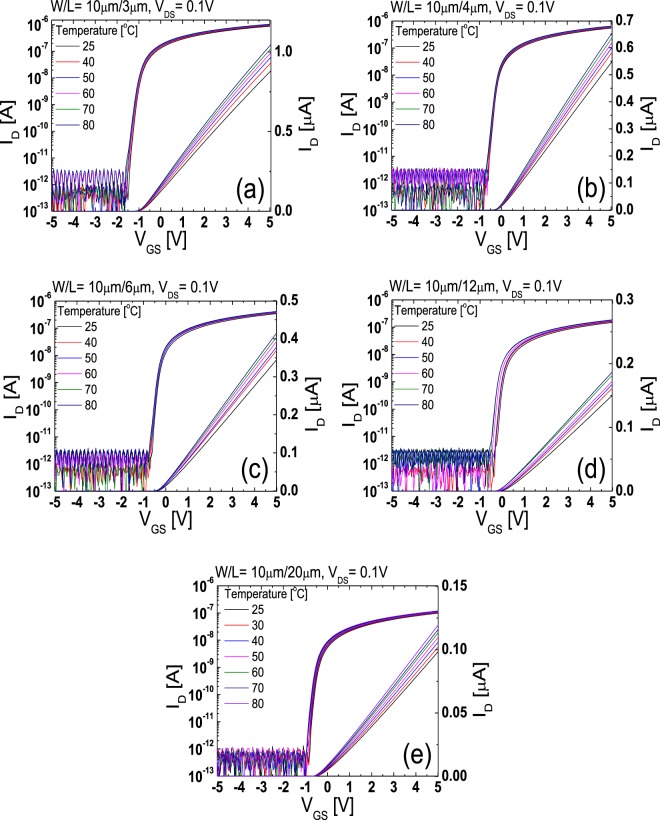
Temperature-dependent linear-mode transfer curves (*V*_DS_ = 0.1 V) measured from the TFTs with different *L*s ((**a**) 3 μm, (**b**) 4 μm, (**c**) 6 μm, (**d**) 12 μm, and (**e**) 20 μm).

**Figure 7:**
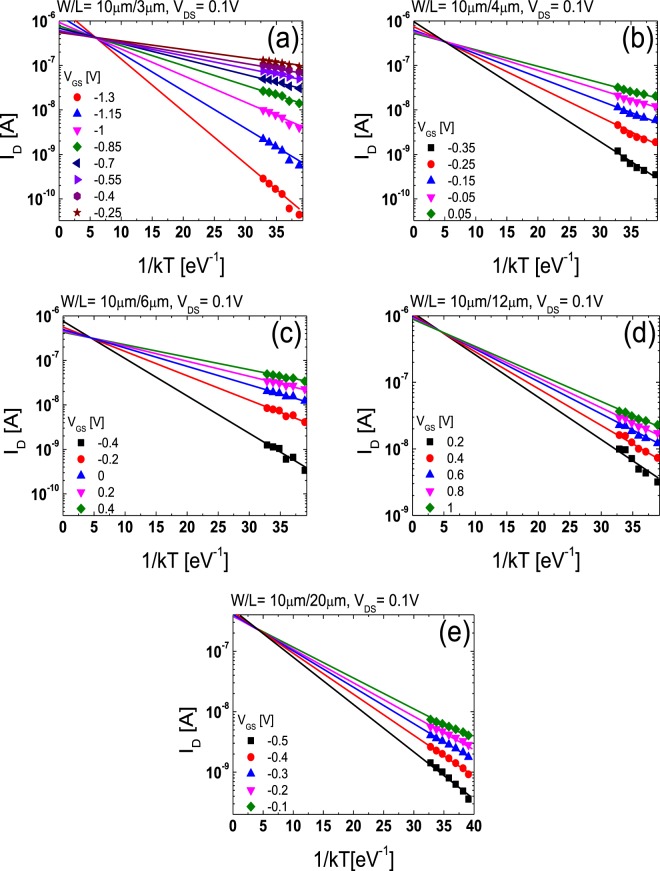
Arrhenius plots obtained from the results in Fig. 6 for TFTs with different *L*s ((**a**) 3 μm, (**b**) 4 μm, (**c**) 6 μm, (**d**) 12 μm, and (**e**) 20 μm).

**Figure 8:**
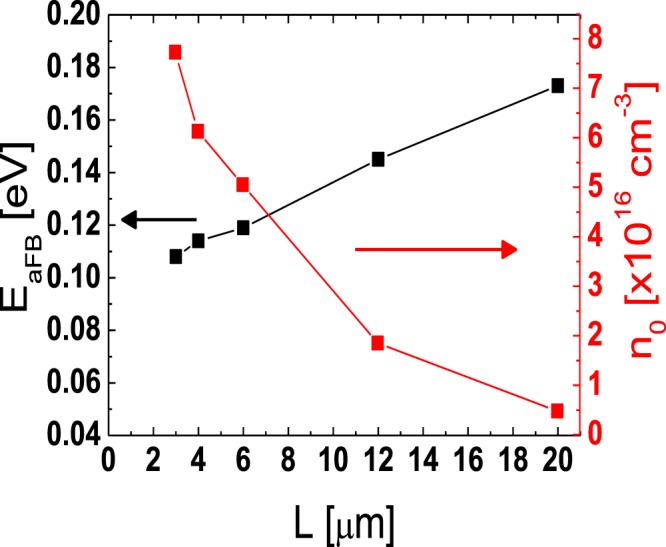
*E*_aFB_ (=*E*_C_ − *E*_F0_) and *n*_0_ extracted from the TFTs with different *L*s.

**Figure 9:**
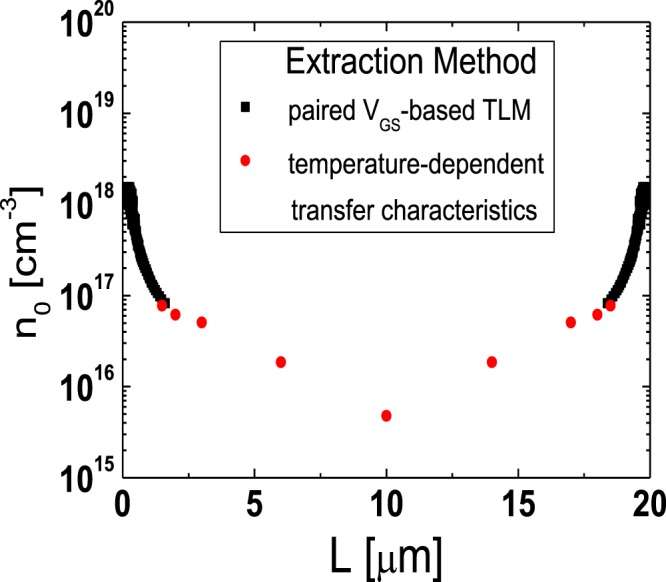
Lateral distribution of *n*_0_ in the whole channel region of the fabricated TG-SA coplanar TFT with *L* = 20 μm.

The lateral carrier concentration distribution in the channel region far from the source/drain junctions can be extracted using the temperature-dependent transfer characteristics data obtained from the TFTs with different *L*s. Figure 6(a–e) show the temperature-dependent linear-mode transfer curves (*V*_DS_ = 0.1 V) of the TFTs with various *L*s (*L* = 3, 4, 6, 12, 20 μm) measured at low *V*_GS_s and Fig. 7(a–e) depict the Arrhenius plots obtained from the results in Fig. 6(a–e). In the disordered semiconductor-based TFTs, the energy distance between the Fermi level and conduction band edge in the flat-band condition (*E*_aFB_ = *E*_C_ − *E*_F0_) has been successfully extracted using the temperature-dependent transfer characteristics according to the procedure described in the previous works^22–24^. Figure 8 shows the evolution of *E*_aFB_ and *n*_0_ extracted from the TFTs with different *L*s using the experimental results in Figs 6 and 7. *n*_0_ was calculated from

3 $${n}_{0}={n}_{{\rm{IGZO}}}\cdot \exp (-{E}_{{\rm{aFB}}}/kT)$$

where *n*_IGZO_ is the effective density of states at the conduction band edge in IGZO at room temperature (=5.0 × 10^18^ cm^−3^)^25^ and *k* is the Boltzmann constant, respectively. Figure 8 shows that *E*_aFB_ increases and *n*_0_ decreases, with an increase in *L*. These results are consistent with the positive shift of *V*_th_ with an increase in *L* observed in Fig. 2. In the TG-SA coplanar TFT, *n*_0_ is different depending on the distance from the edge of the channel due to the carrier diffusion from the n^+^-doped source/drain extension regions. Considering that the carrier diffusion takes place from both source and drain regions, *n*_0_ is believed to have the lowest value in the middle of the channel. Because the *V*_th_ of the laterally non-uniformly doped TFT is determined by the lowest carrier concentration in the channel region, the calculated *n*_0_s in Fig. 8 based on the results in Figs 6 and 7 can be assumed to be the *n*_0_ in the of the middle of the channel in each TFT with different *L*s. These results allow us to extract the values of *n*_0_ as a function of the distance from the edge of the channel in the channel region far from the source/drain junctions. Figure 9 shows the lateral distribution of *n*_0_ in the whole channel region of the fabricated TG-SA coplanar TFT with *L* = 20 μm.

**Figure 10:**
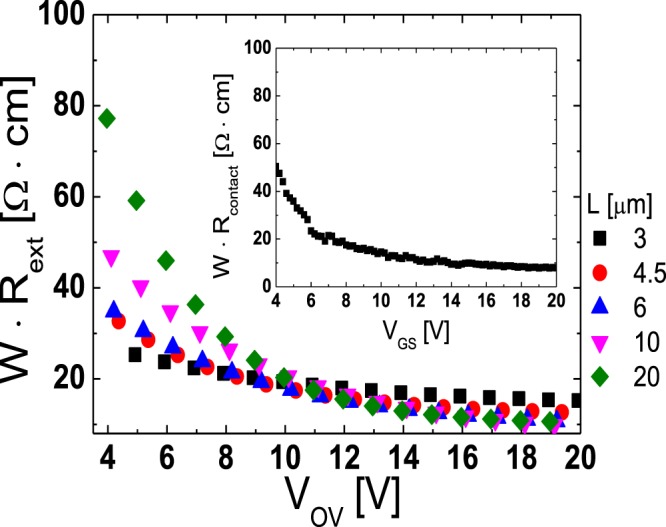
*W* · *R*_ext_ (*W* · (*R*_ext,S_ + *R*_ext,D_)) extracted as a function of *V*_OV_ in the fabricated TG-SA coplanar a-IGZO TFT with different *L*s. For comparison, *W* · *R*_ext_ extracted using the paired *V*_GS_-based TLM in Fig. 4(b) is included as an inset.

Because the relatively higher *R*_ext_ has been considered as a weakness of the TG-SA coplanar structure than the bottom gate structures in a-IGZO TFTs, the extraction of the exact values of *R*_ext_ is very important in the TG-SA coplanar a-IGZO TFTs to further improve the electrical performance of the devices. In this work, we investigated the effects of *L* on the *R*_ext_ of the TG-SA coplanar a-IGZO TFT for the first time using the DCCM. As given in Fig. 4(b), the *R*_ext_ of the TG-SA coplanar a-IGZO TFT can be extracted not only by the DCCM but by the paired *V*_GS_-based TLM. However, because the *R*_ext_s are assumed to be the same in all TFTs with different *L*s in the paired *V*_GS_-based TLM, the extracted *R*_ext_ from the paired *V*_GS_-based TLM is the averaged one of the TFTs with different *L*s. The DCCM was developed to extract the *V*_GS_-induced source and drain series resistance (*R*_ext,S_ and *R*_ext,D_) in the lightly-doped-drain metal-oxide-semiconductor field-effect transistors^26^. It can extract the *R*_ext,S_ and *R*_ext,D_ separately by using the *I*_D_s and output conductances (*G*_D_s) measured in the forward and reverse operation modes in the linear operation regime, respectively. DCCM is to form four independent equations to solve *R*_ext,S_, *R*_ext,D_, *μ*_FE,fwd,_ and *μ*_FE,rev_, where *μ*_FE,fwd_ and *μ*_FE,rev_ are *μ*_FE_s for the forward and reverse mode operations, respectively. Equations (4) and (5) are the two of the four equations which are for the forward mode operation and equations (6) and (7) are those for the reverse mode operation.

4 $${I}_{D}={\mu }_{FE,fwd}\cdot \frac{{C}_{i}\cdot W}{{L}_{eff}}\cdot ({V}_{GS}^{\ast }-{V}_{th}-\frac{1}{2}{V}_{DS}^{\ast })\cdot {V}_{DS}^{\ast }$$

where $${V}_{{\rm{GS}}}^{\ast }$$ = *V*_GS_ − *I*_D_ · *R*_ext,S_ and $${V}_{{\rm{DS}}}^{\ast }$$ = *V*_DS_ − *I*_D_ · (*R*_ext,D_ + *R*_ext,S_).

5 $${G}_{D}=\frac{\partial {I}_{D}}{\partial {V}_{DS}}={\mu }_{FE,fwd}\cdot \frac{{C}_{i}\cdot W}{{L}_{eff}}\cdot [({V}_{GS}^{\ast }-{V}_{th}-\frac{1}{2}{V}_{DS}^{\ast })\cdot \frac{\partial {V}_{DS}^{\ast }}{\partial {V}_{DS}}\,+(\frac{\partial {V}_{GS}^{\ast }}{\partial {V}_{DS}}-\frac{1}{2}\cdot \frac{\partial {V}_{DS}^{\ast }}{\partial {V}_{DS}})\cdot {V}_{DS}^{\ast }]$$

where ∂$${V}_{{\rm{GS}}}^{\ast }$$*/*∂*V*_DS_ = −*G*_D_ · *R*_ext,S_ and ∂$${V}_{{\rm{DS}}}^{\ast }$$*/*∂*V*_DS_ = 1 − *G*_D_ · (*R*_ext,D_ + *R*_ext,S_).

6 $${I}_{D}={\mu }_{FE,rev}\cdot \frac{{C}_{i}\cdot W}{{L}_{eff}}\cdot ({V}_{GS}^{\ast }-{V}_{th}-\frac{1}{2}{V}_{DS}^{\ast })\cdot {V}_{DS}^{\ast }$$

where $${V}_{GS}^{\ast }$$ = *V*_GS_ *−* *I*_D_ · *R*_ext,D_ and $${V}_{DS}^{\ast }$$ = *V*_DS_ − *I*_D_ · (*R*_ext,D_ + *R*_ext,S_).

7 $$\begin{array}{c}{G}_{D}=\frac{\partial {I}_{D}}{\partial {V}_{DS}}={\mu }_{FE,rev}\cdot \frac{{C}_{i}\cdot W}{{L}_{eff}}\cdot [({V}_{GS}^{\ast }-{V}_{th}-\frac{1}{2}\cdot {V}_{DS}^{\ast })\cdot \frac{\partial {V}_{DS}^{\ast }}{\partial {V}_{DS}}\\ \,\,+(\frac{\partial {V}_{GS}^{\ast }}{\partial {V}_{DS}}-\frac{1}{2}\cdot \frac{\partial {V}_{DS}^{\ast }}{\partial {V}_{DS}})\cdot {V}_{DS}^{\ast })]\end{array}$$

where ∂${V}_{GS}^{\ast }$*/*∂*V*_DS_ = −*G*_D_ · *R*_ext,D_ and ∂${V}_{{\rm{DS}}}^{\ast }$*/*∂*V*_DS_ = 1 − *G*_D_ · (*R*_ext,D_ + *R*_ext,S_). *R*_ext,S_, *R*_ext,D_, *μ*_FE,fwd_, and *μ*_FE,rev_ can be extracted from the measured forward and reverse mode *I*_D_ and *G*_D_ at any specified *V*_GS_ by solving the four equations simultaneously by numerical methods. Because the DCCM requires only a single device for the extraction of *R*_ext_, it can be used to investigate the effects of *L* on the *R*_ext_ in the TG-SA coplanar a-IGZO TFTs. Figure 10 shows the *W* · *R*_ext_ (*W* · (*R*_ext,S_ + *R*_ext,D_)) extracted as a function of *V*_OV_ in the fabricated TG-SA coplanar a-IGZO TFT with different *L*s, where *V*_OV_ represents *V*_GS_ − *V*_th_. For comparison, the *W* · *R*_ext_ extracted using the paired *V*_GS_-based TLM in Fig. 4(b) is also included as an inset. The results in Fig. 10 clearly shows that the *R*_ext_ is increased with an increase in *L* especially at low *V*_GS_s. Considering that the *R*_ext_ at low *V*_GS_s is dominated by the unintentionally doped channel regions formed by the lateral carrier diffusion from the n^+^-doped source/drain extension regions near the source/drain junctions, the higher *R*_ext_ in the longer channel TFTs is believed to be mainly caused by the enhanced carrier diffusion (large Δ*L*) due to the larger gradient of the carrier concentration in the longer channel devices.

## Conclusion

In this work, we examined the lateral distribution of *n*_0_ across the channel and the effects of *L* on the *R*_ext_ in the TG-SA coplanar a-IGZO TFTs. The lateral distribution of *n*_0_ across the channel was extracted using the paired *V*_GS_-based TLM near the source/drain junctions and using the temperature-dependent transfer characteristics data measured from the TFTs with different *L*s near the middle of the channel, respectively. *n*_0_ abruptly decreased from 1.5 × 10^18^ cm^−3^ to 8.2 × 10^16^ cm^−3^ at room temperature as the distance from the edge of the channel (Δ*L*/2) increases from 0.16 μm to 1.63 μm in the fabricated TG-SA coplanar TFT with *L* = 20 μm. However, much smaller gradient of *n*_0_ was observed in the channel region far from the source/drain junctions. To examine the effect of *L* on the *R*_ext_ in the TG-SA coplanar a-IGZO TFTs, the DCCM was employed. The *R*_ext_s were extracted from the TFTs with different *L*s, which clearly showed that *R*_ext_ increased with an increase in *L* especially at low *V*_GS_s. The observed phenomenon was possibly attributed to the enhanced carrier diffusion (large Δ*L*) near the source/drain junctions in the long channel devices.

## Methods

An a-IGZO layer (In:Ga:Zn = 1:1:1 at%) was deposited by direct-current sputtering at room temperature on a SiO_2_ buffered glass substrate. A SiO_2_ layer was deposited by plasma-enhanced chemical vapor deposition as a gate insulator followed by the sequential deposition of a gate metal. After deposition and patterning of the gate electrode and the gate insulator, the source/drain extension regions were self-aligned to the gate and are n^+^-doped by being exposed to the plasma during the dry-etching process. Interlayer dielectrics were deposited and patterned for source/drain contact holes. Then, the metal layer was sputtered and patterned to form the source/drain electrodes. The TFTs were passivated by a SiO_2_ passivation layer. Finally, the devices were thermally annealed to achieve the stable and uniform electrical performances.

## References

[1] Nomura K (2004). Room Temperature Fabrication of Transparent Flexible Thin-film Transistors Using Amorphous Oxide Semiconductors. Nature.

[2] Kamiya T, Hosono H (2010). Material Characteristics and Applications of Transparent Amorphous Oxide Semiconductors. NPG Asia Mater..

[3] Kwon J-Y, Lee D-J, Kim K-B (2011). Transparent Amorphous Oxide Semiconductor Thin Film Transistor. Electronic Materials Letters.

[4] Lee SY, Kim DH, Chong E, Jeon YW, Kim DH (2011). Effect of Channel Thickness on Density of States in Amorphous InGaZnO Thin Film Transistor. Appl. Phys. Lett..

[5] Fortunato E, Barquinha P, Martins R (2012). Oxide Semiconductor Thin-film Transistors: A Review of Recent Advances. Adv. Mater.

[6] Bak JY (2015). Origin of Degradation Phenomenon under Drain Bias Stress for Oxide Thin Film Transistors using IGZO and IGO Channel Layers. Sci. Rep..

[7] Kwon JY, Jeong JK (2015). Recent Progress in High Performance and Reliable N-type Transition Metal Oxide-based Thin Film Transistors. Semicond. Sci. Technol..

[8] Kim Y-H, Lee E, Um JG, Mativenga M, Jang J (2016). Highly Robust Neutral Plane Oxide TFTs Withstanding 0.25 mm Bending Radius for Stretchable Electronics. Sci. Rep..

[9] Yang S (2011). Low-Temperature Processed Flexible In-Ga-Zn-O Thin-film Transistors Exhibiting High Electrical Performance. IEEE Electr. Device Lett..

[10] Kim W-G (2016). High-pressure Gas Activation for Amorphous Indium-Gallium-Zinc-Oxide Thin-Film Transistors at 100 °C. Sci. Rep..

[11] Osada T (2010). Development of Liquid Crystal Display Panel Integrated with Drivers Using Amorphous In-Ga-Zn-Oxide Thin Film Transistors. Jpn. J. Appl. Phys..

[12] Yoon J-S (2014). 55-inch OLED TV using Optimal Driving Method for Large-size Panel based on InGaZnO TFTs. SID Int. Symp. Dig. Tech. Pap..

[13] Oh S (2014). Comparison of Top-Gate and Bottom-Gate Amorphous InGaZnO Thin-film Transistors with the Same SiO_2_/a-InGaZnO/SiO_2_ Stack. IEEE Electr. Device Lett..

[14] Choi S (2017). Systematic Decomposition of the Positive Bias Stress Instability in Self-aligned Coplanar InGaZnO Thin-Film Transistors. IEEE Electr. Device Lett..

[15] Bae JU (2013). Development of Oxide TFT’s Structures. SID Int. Symp. Dig. Tech. Pap..

[16] Jang YH (2017). Internal Compensation Type OLED Display Using High Mobility Oxide TFT. SID Int. Symp. Dig. Tech. Pap..

[17] Kang DH, Han JU, Mativenga M, Ha SH, Jang J (2013). Threshold Voltage Dependence on Channel Length in Amorphous-Indium-Gallium-Zinc-Oxide Thin-Film Transistors. Appl. Phys. Lett..

[18] Ha SH (2013). Channel Length Dependent Bias-Stability of Self-Aligned Coplanar a-IGZO TFTs. J. Display Technol..

[19] Kim HW, Kim ES, Park JS, Lim JH, Kim BS (2018). Influence of Effective Channel Length in Self-aligned Coplanar Amorphous-Indium-Gallium-Zinc-Oxide Thin-film Transistors with Different Annealing Temperatures. Appl. Phys. Lett..

[20] Hu GJ, Chang C, Chia Y-T (1987). Gate-Voltage-Dependent Effective Channel Length and Series Resistance of LDD MOSFET’s. IEEE Trans. Electron Devices..

[21] Chern HN, Lee CL, Lei TF (1995). An Analytical Model for the Above-threshold Characteristics of Polysilicon Thin-film Transistors. IEEE Trans. Electron Devices..

[22] Chen C, Abe K, Kumomi H, Kanicki J (2009). Density of States of A-InGaZnO from Temperature-dependent Field-effect Studies. IEEE Trans. Electron Devices..

[23] Jeong J, Jeong JK, Park J-S, Mo Y-G, Hong Y (2010). Meyer-Neldel Rule and Extraction of Density of States in Amorphous Indium-Gallium-Zinc-Oxide Thin-Film Transistor by Considering Surface Band Bending. Jpn. J. Appl. Phys..

[24] Jeong C-Y, Lee D, Han Y-J, Choi Y-J, Kwon H-I (2015). Subgap States in P-channel Tin Monoxide Thin-film Transistors from Temperature-dependent Field-effect Characteristics. Semicond. Sci. Technol..

[25] Fung T-C (2009). Two-dimensional Numerical Simulation of Radio Frequency Sputter Amorphous In-Ga-Zn-O Thin-film Transistors. J. Appl. Phys..

[26] Lou C-L, Chim W-K, Chan DS-H, Pan Y (1998). A Novel Single-device DC Method for Extraction of the Effective Mobility and Source-drain Resistances of Fresh and Hot-carrier Degraded Drain-engineered MOSFET’s. IEEE Trans. Electron Devices..

